# An Explainable Multimodal Deep‐Learning Model Using Histopathological Image and Clinical Information for Prognosis Prediction in Colorectal Cancer: A Multi‐Center Cohort Study

**DOI:** 10.1002/advs.78417

**Published:** 2026-10-26

**Authors:** Zhihan Jiang, Xiangchao Shi, Chenghan Su, Yao Ma, Rui Li, Menglei Chen, Jianxin Cui, Nicholas Hawkins, Robyn Ward, Xiangzhi Bai, Jitendra Jonnagaddala, Wei Fu, Xin Zhou

**Affiliations:** ^1^ Department of General Surgery, Peking University Third Hospital Peking University Beijing People's Republic of China; ^2^ Peking University Third Hospital Cancer Center Peking University Beijing People's Republic of China; ^3^ Beijing Key Laboratory for Interdisciplinary Research in Gastrointestinal Oncology (BLGO) Beijing People's Republic of China; ^4^ Department of General Surgery, The First Medical Center Chinese PLA General Hospital Beijing People's Republic of China; ^5^ School of Biomedical Sciences, Faculty of Medicine and Health UNSW Sydney Kensington Australia; ^6^ Office of DVCR Monash University Melbourne Australia; ^7^ Image Processing Center Beihang University Beijing People's Republic of China; ^8^ State Key Laboratory of Virtual Reality Technology and Systems Beihang University Beijing People's Republic of China; ^9^ Key Laboratory of Spacecraft Design Optimization and Dynamic Simulation Technology, Ministry of Education Beihang University Beijing People's Republic of China; ^10^ School of Clinical Medicine, Faculty of Medicine and Health UNSW Sydney Kensington Australia; ^11^ SREDH Consortium Sydney Australia

**Keywords:** colorectal cancer, computational pathology, deep learning, multimodal fusion, prognosis prediction

## Abstract

Accurate prognostication is crucial for the individualized management of patients with colorectal cancer (CRC), while the accuracy and interpretability of current models still need to be improved. This study aims to develop and validate an explainable multimodal model for precise survival prediction. 3381 patients with resected CRC are enrolled from three medical centers, and a multimodal deep‐learning framework integrating clinical variables and histopathological images is developed. The multimodal model exhibits significantly more robust performance than unimodal models in prognosis prediction across all of the cohorts. By stratifying patients into low‐risk and high‐risk groups based on the model‐derived risk scores, high‐risk patients exhibit worse outcomes, and the stratification remains an independent prognostic factor in multivariable Cox regression. The model also identifies patients with markedly poorer survival among the stage II/III subgroup. Adjuvant chemotherapy demonstrates benefits in high‐risk patients with stage III CRC but not in the low‐risk group. Multi‐omic analyses disclose that low‐risk tumors are characterized by enhanced adaptive immune responses, whereas high‐risk tumors exhibit stronger angiogenesis and higher exhaustion scores of T cells, reflecting a hypoxic and immunosuppressive microenvironment. These results support the potential of the multimodal model to effectively predict survival outcomes in resected CRC with great explainability.

## Graphical abstract

This study develops a multimodal deep‐learning model that integrates clinical variables and histopathological images to predict survival outcomes in colorectal cancer. The multimodal model effectively stratifies patients into low‐ and high‐risk groups, demonstrating superior accuracy and generalizability across multiple independent cohorts. Further multi‐omic analyses delineate distinct molecular features across risk groups, enhancing the biological interpretability.

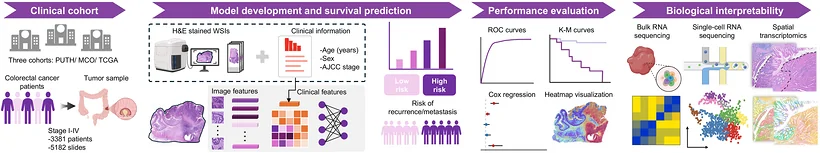

## 1 Introduction

Colorectal cancer (CRC) is the third most common malignancy with the second‐highest cancer‐related deaths worldwide, posing a significant economic and social burden [1]. Despite advances in early detection and surgical techniques, post‐operative recurrence remains a major clinical issue for CRC, so adjuvant chemotherapy (ACT) is widely recommended to reduce the risk of recurrence in stage III CRC [2, 3, 4]. Effective risk stratification is essential for optimizing individualized treatment strategies, considering the toxicity and cost of these therapies [5, 6, 7]. In clinical practice, CRC treatment is predominantly guided by TNM staging, which is insufficient to capture the biological complexity of tumor and has notable limitations on individual‐level prognosis prediction [8, 9]. Therefore, a broadly accessible and reliable technique for the accurate prognostication of CRC is urgently needed.

Hematoxylin and eosin (H&E)‐stained histopathological slides are the gold standard for CRC diagnosis and contain extensive information on tumor morphology and its microenvironment, which may serve as an innovative prognostic biomarker. Advancements in deep learning, coupled with progress on computational histopathology, have made remarkable strides in prognosis prediction based on histopathological whole‐slide images (WSIs) [10]. Recent researches have demonstrated the potentials of WSIs to predict survival in CRC patients [11, 12, 13]. Whereas, utilization of unimodal data constrains the comprehensiveness of analysis in this field and hinders broadly clinical application due to absence of generalization [14]. Clinical variables, which include essential information of patients and lesions, are routinely collected in practice and can serve as complements to histopathological WSIs by enhancing the model's perception to overall context [15]. By leveraging attention‐based fusion methods, multimodal models can acquire cross‐modal interactions from diverse perspectives and facilitate an effective feature fusion [16]. Multimodal approaches that adequately incorporate clinical information and WSIs have offered new opportunities to improve predictive performance and optimize individual risk stratification for CRC. Several studies have used such patterns and highlighted its considerable performance, nevertheless, there are certain limitations including insufficient sample size of external validation and incomplete analysis of clinical benefits from adjuvant treatment, as well as lack of explainability or interpretability [17].

To address the aforementioned limitations, we collected data that comprised clinical variables and histopathological WSIs across three independent clinical cohorts with 3381 patients with CRC and proposed a multimodal deep‐learning model for accurate prognosis prediction in this study. Based on an advanced self‐supervised foundational model, an attention‐based multiple‐instance learning (ABMIL) algorithm, and a cross‐attention fusion architecture (Figure 1a,b), we validated the improved robustness and generalizability of our model on internal and external datasets, highlighting its reliability as a tool for guiding personalized treatment. Additionally, we integrated multi‐omic sequencing data, including bulk RNA, single‐cell RNA (scRNA), and spatial transcriptomics, to deeply investigate the biological mechanisms underlying low‐risk and high‐risk groups from our model (Figure 1c), aimed to enhance its interpretability at the molecular level.

**FIGURE 1:**
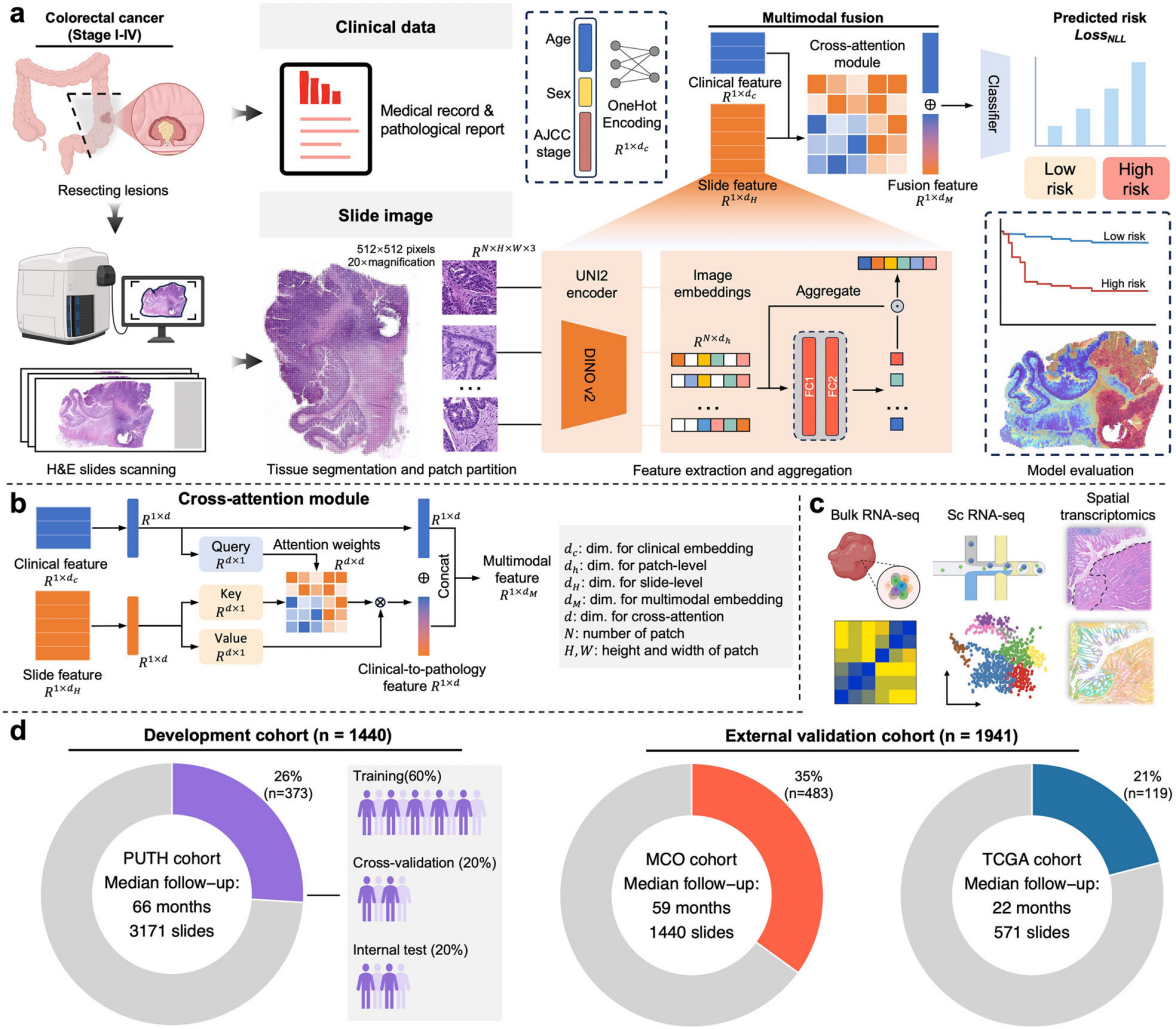
The overall study design. (a) Methodological workflow of this study. Clinical variables (age, sex, and AJCC stage) and digital histopathological images are acquired as inputs of the multimodal deep‐learning model. Clinical data are encoded by One‐hot encoding and MLP layers to yield clinical embeddings. Patch‐level features of slides are extracted by the UNI2 foundation model and further aggregated into slide‐level features using the attention‐based multiple instance learning method, followed by a cross‐attention module for comprehensive fusion with clinical embeddings. The negative log‐likehood loss is employed for prognosis prediction over discrete time, and risk scores are defined as the integrated predicted probabilities. (b) Details of the cross‐attention module. (c) Biological interpretability. (d) Composition of patient cohort. A total of 3381 eligible patients with colorectal cancer are enrolled for model development and evaluation. Abbreviations: FC, fully connected layer. H&E, hematoxylin and eosin. MCO, Molecular and Cellular Oncology. MLP, multilayer perceptron. NLL, negative log‐likehood. PUTH, Peking University Third Hospital. Sc RNA‐seq, single‐cell RNA sequencing. TCGA, The Cancer Genome Atlas.

## 2 Methods

### 2.1 Populations and Study Cohorts

In this retrospective study, we enrolled a total of 3381 patients with CRC harboring complete clinical information and H&E‐stained histopathological slides across three international cohorts: the Peking University Third Hospital cohort (PUTH, n = 1440, 3171 slides), the Molecular and Cellular Oncology study (MCO, n = 1370, 1440 slides) [18, 19, 20], and The Cancer Genome Atlas dataset (TCGA, n = 571, 571 slides), which were from China, Australia, and America, respectively (Figure 1d). All participants underwent surgical resection without neoadjuvant chemoradiotherapy and had a diagnosis of colorectal adenocarcinoma confirmed by postoperative pathology. Patients without complete clinical information or high‐quality H&E slides were excluded. Details about patient enrollment were shown in Figure S1 and Table S1. Data from the PUTH cohort were used for model training, and another two cohorts were used for external validation. In addition, tumor samples of 111 and 21 CRC patients from PUTH were collected for bulk RNA and scRNA sequencing, respectively, to explore tumor biological signatures associated with prognosis prediction (Table S1). All data were fully anonymized at source before analysis. TRIPOD+AI statement was followed to report these results [21].

Survival data in this study included disease‐specific disease (DSS), overall survival (OS), and disease‐free survival (DFS). Follow‐up was conducted through outpatient visits or phone calls from the cancer registry, including information about tumor recurrence, progression, and mortality. DFS was only available for the PUTH cohort. The primary outcome of this study was DSS, defined as the interval from the date of initial surgery to death due to CRC progression. All research procedures adhered to the principles of the Declaration of Helsinki, and the study was approved by the Peking University Third Hospital Ethical Review Board (IRB00006761‐M2024534). Informed consent was waived for the retrospective components, whereas written consent was obtained from patients who contributed tumor specimens for bulk RNA and scRNA sequencing.

### 2.2 Slide Digitization and Data Preprocessing

All formalin‐fixed paraffin‐embedded (FFPE) tumor sections were stained with H&E and digitized as WSIs using high‐resolution digital slide scanners at respective institutions (Figure 1a). Details of slide scanner and scanning parameters are presented in Table S1. All WSIs in this study were preprocessed using the Trident toolkit (https://github.com/mahmoodlab/TRIDENT). Specifically, tissue area of the WSI was first segmented by a DeeplabV3+‐based segmentation model. To address the resolution heterogeneity across cohorts, all WSIs were harmonized to an equivalent magnification of 20×. For WSIs originally scanned at 20×, nonoverlapping patches of 512×512 pixels were directly extracted. For those scanned at 40×, patches of 1024 × 1024 pixels were extracted and down‐sampled to 512×512 pixels, yielding patches that represented the same physical tissue area. Only patches with at least 70% tissue coverage were retained, and their coordinates were saved for the following pipeline. Macenko method was subsequently applied for the staining normalization of all patches [22]. To extract meaningful image representations from WSI, we employed a Vision Transformer‐based pretrained encoder, UNI2, which converted each patch image into a 1536‐dimensional feature vector [23]. For each WSI, a feature vector matrix of N×1536‐dimension was generated by concatenating patch feature within it, where N denoted the total number of patches per WSI.

Clinical data of PUTH were retrieved from digital medical records and pathological reports (Figure 1a). To reduce redundancy of multimodal information and expand application scenarios, only three core clinical variables were collected: age (years, continuous), sex (male or female, categorical), and American Joint Committee on Cancer (AJCC) stage (categorical) [24]. After standardization and one‐hot encoding procession, clinical variables of each patient were converted into a 6‐dimensional embedding for multimodal fusion.

### 2.3 Multimodal Deep Learning Model Framework

Our multimodal deep learning model employed a weakly supervised deep learning model and a cross‐attention transformer module to effectively incorporate clinical and histopathological information and enhance prognosis prediction (Figure 1a,b). First, to aggregate patch‐level features, we adopted the ABMIL as backbone [25]. In particular, assuming that $h\in R^{N\times 1536}$ is the input feature matrix representing a WSI with *N* patches, this approach applied linear neural network to output an attention score *a_n_* for the *n*‐th patch and calculated the weighted average of patches to generate a slide‐level embedding $H\in R^{1\times 1536}$:

$$\;a_{n}=\frac{\exp\left\{w^{⊤}\left(\tanh\left(Vh_{n}^{⊤}\right)⊙\mathrm{sigm}\left(Uh_{n}^{⊤}\right)\right)\right\}}{\sum_{j=1}^{N}\exp\left\{w^{⊤}\left(\tanh\left(Vh_{j}^{⊤}\right)⊙\mathrm{sigm}\left(Uh_{j}^{⊤}\right)\right)\right\}}$$

$$H=\sum_{n=1}^{N}a_{n}h_{n}$$

where $w\in R^{L\times 1}$, $V\in R^{L\times 1536}$, and $U\in R^{L\times 1536}$ are trainable parameters, with L being the hidden attention dimension.

Then, we introduced a unidirectional cross‐attention module to capture cross‐modal interactions and fulfill feature fusion. To map the embeddings of each modality to the same dimension, clinical ($C\in R^{1\times 6}$) and pathological ($H\in R^{1\times 1536}$) embeddings were projected into a common latent feature space of dimension *d*  =  512 by two linear encoders, respectively, obtaining $X_{C}\in R^{1\times d}$ and $X_{H}\in R^{1\times d}$. Subsequently, the cross‐attention layer computed a query‐key product in the following way to extract the clinically relevant histopathological embedding $F_{C\to H}$:

$$Q=X_{C}^{⊤}\;W_{Q},\;K=X_{H}^{⊤}\;W_{K},\;V=X_{H}^{⊤}\;W_{V}$$

$$A=\mathrm{softmax}\left(\frac{QK^{⊤}}{\sqrt{d_{k}}}\right)$$

$$\;F_{C\to H}={\left(A\cdot V\right)}^{⊤}$$

where ***W**_Q_*, ***W**_K_*, ***W**_V_* were learnable parameters, resulting in $Q,\;K,\;V\in R^{d\times 1}$. The attention map $A\in R^{d\times d}$ was computed by the product of *Q* and *K*, followed by a softmax operation. The final multimodal embedding $F_{multimodal}\in R^{1\times 1024}$ was formed by concatenating projected clinical embedding *X_C_* and $F_{C\to H}$:

$$\;F_{multimodal}=\mathrm{Concat}\left(X_{C},\;F_{C\to H}\right)$$

Lastly, for prognosis prediction, we adopted the negative log‐likehood (NLL) loss function considering the deviation caused by censor rate, which transformed survival prediction into a classification task by prediction the probability that survival time fell into different time interval [26]. Disease‐specific survival time were divided into four nonoverlapping intervals: [t_0_, t_1_), [t_1_, t_2_), [t_2_, t_3_), [t_3_, t_4_), where t_0_ = 0, t_4_ = ∞, and t_1_, t_2_, t_3_ defined the quartiles of the event times for non‐censored patients. Let *k* (k∈{1,2,3,4}) be the interval index in which the ground‐truth event or censoring occurred for a given patient. The model output a discrete hazard vector H  =  [*h*_1_, *h*_2_, *h*_3_, *h*_4_] to estimate the conditional probability of death within the respective interval. Consequently, the patient‐level survival probability function *S*(*k*), which represented the probability of surviving past interval *k*, was defined as:

$$S\;\left(k\right)=\prod_{i=1}^{k}\left(1-h_{i}\right)$$

For each patient, let c∈{0,1} denoted the event indicator, where *c*  =  0 indicated the occurrence of the disease‐specific event during follow‐up, and *c*  =  1 indicated that the patient was alive at the last follow‐up record (right‐censored). To jointly optimize for both censored and uncensored patients, the NLL loss was calculated as:

$$\;L_{survival}=-\left(1-c\right)\log\left(S\left(k-1\right)\cdot h_{k}\right)-c\log\left(S\left(k\right)\right)$$

### 2.4 Experimental Design and Statistics Analysis

For model development and evaluation, we performed all experiments using fivefold Monte Carlo cross‐validation. An internal test dataset (20% of cases) was first held out from PUTH dataset, and the remaining data were randomly split into training (60%) and validation (20%) sets in each fold, stratified by each class of AJCC stage. Given that participants might have several slides, slides from the same participant were sampled into one set of train/validation/test to avoid information leakage. The models were further examined using external datasets. Implementation details of model development were shown in Table S2. To evaluate the model's prediction performance, concordance index (C‐index) and the areas under the time‐dependent receiver operating characteristic curves (AUC, at 3‐year and 5‐year) for DSS, OS, and DFS were calculated. The best model checkpoint with the highest C‐index on validation sets was applied for survival analysis. Kaplan–Meier (K–M) curves, log‐rank tests, and Cox proportional hazard regression were implemented to investigate the survival differences between the low‐risk and high‐risk patients, and the cutoff (0.366) was determined using the 60th percentile of predicted scores derived from the training set. We compared the prediction performance of the proposed model with other unimodal or multimodal methods, and details can be found in Table S2.

Model training and test were accomplished based on PyTorch (version 2.8.0). All data were analyzed using R software (version 4.3.3) and Python (version 3.10.18). For continuous variables, medians with interquartile ranges (IQR) were presented, and differences were calculated using Kruskal–Wallis test. For categorical variables, numbers with percentages were presented, and differences were calculated using χ^2^ or Fisher's exact test. C‐index calculation, K–M curves, log‐rank tests, and Cox regression were conducted using R packages *survival* and *survminer*. Time‐dependent ROC analysis was conducted by using R package *timeROC* and visualized using Python library *scikit‐learn*. A two‐sided P‐value < 0.05 was considered statistically significant. Source code is available at: https://github.com/Revol2Revol/SurvCRC.

### 2.5 Heatmap Visualization and Model Explainability

To reveal high‐attention regions of histopathological WSIs that contribute to survival prediction, WSI‐level attention heatmap visualization was conducted by mapping attention scores of patches to corresponding spatial locations. The extracted patch embeddings of specific WSI were passed through multimodal model for inference, and the attention score for each patch was collected. These attention scores were normalized to a range between 0 and 1, where a higher score corresponded to a more attentive distribution. The normalized scores were arranged according to their corresponding coordinates on original WSIs, resulting in the attention heatmaps. Additionally, we extracted the high‐attention regions of interest (ROIs) and applied HoverNet to fulfill simultaneous cell segmentation and type summarization to grain further insights into the tumor microenvironment [27]. Cell fraction distribution on high‐attention ROIs was subsequently compared between low‐risk and high‐risk patients.

### 2.6 Multi‐Omic Data Analysis

To explore the biological explainability of the risk score, we analyzed the bulk RNA and scRNA sequence data from the PUTH cohort and publicly available high‐resolution spatial transcriptomic data from the 10×Genomics Visium HD and Xenium spatial gene expression dataset portal (Figure 1c). Importantly, the histopathological images used for analyzing were from FFPE tissues exactly adjacent to the fresh tissues used for bulk RNA and scRNA sequencing, in order to ensure the correspondence between molecular information and pathological information.

A total of 111 tissue samples from PUTH were collected and subjected to bulk RNA sequencing. Patients were stratified into high‐risk and low‐risk groups by applying trained multimodal model to matched histopathological images and clinical data. Differentially expressed genes (DEGs) between the two risk groups were identified using the DESeq2 package in R with stringent criteria (|log2(fold change)| > 1.0 and adjusted p < 0.05), followed by functional annotation of DEGs through pathway enrichment analysis based on Kyoto Encyclopedia of Genes and Genomes (KEGG) and Gene Ontology (GO) databases. Immune infiltration was assessed using MCPcounter algorithm to estimate the relative abundance of immune cell subsets.

Single‐cell RNA sequencing data from resected CRC specimens (n = 21) were further analyzed. Details of all samples were included in Table S3. We used Seurat (v4.3.0) package in R to process the data. For quality control, we removed genes expressed in fewer than 3 cells, and cells with <200 or >6000 genes or >25% mitochondrial genes were filtered, resulting in 158 066 cells retained for downstream analysis. For dimensionality reduction and clustering, we normalized the expression matrix in Seurat and selected 2000 highly variable genes to perform principal component analysis. To eliminate batch effects, we performed batch correction using the Harmony package in R. After scaling the data, the first 30 principal components were computed to cluster cells. We used the *Findneighbours* function in Seurat to obtain the number of nearest neighbors and used the *Findclusters* function for clustering cell subtypes. Next, we set the resolution to 0.5 for all cell clustering. Cells were categorized into epithelial cells, B cells, fibroblast, endothelial cells, mast cells, myeloid cells, neutrophils, natural killer (NK)/T cells, pericytes, and plasma cells based on classical gene markers (Table S4). We then performed a second‐round clustering of cell subpopulation to identify different subpopulations in CRC, with each subtype being named by a highly expressed gene. To assess the function of cell subclusters, we calculated the functional module scores at the single‐cell level using the *AddModuleScore* function in Seurat.

High‐definition spatial transcriptomics datasets of human colorectal cancer were obtained from the 10x Genomics official website. Data importing, quality control, and normalization were performed using R. Subsequently, the top 20 high‐attention regions were annotated based on the deep learning‐derived attention scores. We used the Seurat package in R to calculate and identify the upregulated DEGs within these high‐attention patches. Functional enrichment analysis, including KEGG and GO pathways, was conducted using the clusterProfiler package in R. Finally, the spatial visualization of transcriptomic signatures was generated using Xenium Explorer.

## 3 Results

### 3.1 Study Design and Patient Cohort Characteristics

This study aimed to develop an explainable multimodal deep‐learning model that integrates routinely collected clinical variables and histopathological WSIs for accurate prognosis prediction among patients with CRC, ultimately guiding personalized treatment and improving patient outcomes. To achieve this, we conducted a multicenter discovery and validation cohort study. Overall model pipeline and cohort composition were depicted in Figure 1. A total of 3381 patients with CRC from three retrospective cohorts were included and stratified into training (n = 864), cross‐validation (n = 287), internal test (n = 289), and external validation (n = 1941). The median follow‐up times were 66, 59, and 22 months for PUTH, MCO, and TCGA cohorts, respectively, with death rates shown in Figure 1d. The demographics and clinical characteristics of participants from each cohort were summarized in Table 1. Baseline characteristics were comparable among each set of PUTH cohort (Table S5).

**TABLE 1:** Clinical characteristics of multicenter CRC cohorts in this study.

Characteristic	PUTH (N = 1440)	MCO (N = 1370)	TCGA (N = 571)
**Gender**			
Male	847 (59%)	754 (55%)	292 (51%)
Female	593 (41%)	616 (45%)	279 (49%)
**Age (years)**	66 [58, 75]	69 [60, 77]	68 [58, 76]
**Tumor location**			
Left	533 (37%)	337 (25%)	127 (22%)
Right	416 (29%)	508 (37%)	198 (35%)
Rectum	491 (34%)	522 (38%)	148 (26%)
Unknown	0 (0%)	3 (<1%)	98 (17%)
**T stage**			
T1	148 (10%)	110 (8%)	19 (3%)
T2	308 (21%)	226 (16%)	100 (18%)
T3	805 (56%)	709 (52%)	390 (68%)
T4	179 (12%)	325 (24%)	62 (11%)
**N stage**			
N0	900 (63%)	780 (57%)	322 (56%)
N1	397 (28%)	345 (25%)	140 (25%)
N2	143 (10%)	245 (18%)	109 (19%)
**M stage**			
M0	1363 (95%)	1193 (87%)	490 (86%)
M1	77 (5%)	177 (13%)	81 (14%)
**AJCC stage**			
Stage I	374 (26%)	267 (19%)	103 (18%)
Stage II	505 (35%)	483 (35%)	211 (37%)
Stage III	484 (34%)	443 (32%)	174 (30%)
Stage IV	77 (5%)	177 (13%)	83 (15%)
**Follow‐up duration (months)**	66 [48, 91]	59 [30, 59]	22 [13, 36]
**OS status**	373 (26%)	483 (35%)	117 (20%)
**DSS status**	308 (21%)	329 (24%)	86 (15%)
**DFS status**	436 (30%)	—	—

Data are presented as median (IQR) for continuous variables and number (%) for categorial variables.

### 3.2 Prediction Performance of the Multimodal Model

The prognostic prediction performance of all models was comprehensively evaluated across three cohorts using the C‐index, where a higher C‐index indicated better capacity to rank the survival times. The proposed multimodal model achieved enhanced C‐indexes across all datasets compared with other unimodal and multimodal models. Specifically, for predicting DSS, our model achieved C‐indexes of 0.849 (95% CI, 0.845–0.854) in the internal test, 0.811 (95% CI, 0.807–0.816) in the MCO cohort, and 0.730 (95% CI, 0.724–0.737) in the TCGA cohort (Figure 2a). Performance for OS prediction slightly declined, while our model also demonstrated superior discriminative power, with C‐indexes of 0.830 (95% CI, 0.827–0.832) in the internal test, 0.727 (95% CI, 0.723–0.730) in the MCO cohort, and 0.694 (95% CI, 0.687–0.702) in the TCGA cohort (Figure 2b). A similar level of accuracy was observed for predicting DFS, with C‐indexes of 0.821–0.855 in the PUTH cohort (Figure 2c). The C‐indexes of DSS and OS for non‐metastatic patients in each cohort were shown in Table S6. Distributions of prediction scores derived from each model were presented in Figure S2. The multimodal model‐derived risk scores were significantly associated with survival status across three cohorts (all P‐values < 0.001, Figure 2d–f). The AUCs for predicting 3‐year DSS were 0.879, 0.841, and 0.750 in PUTH, MCO, and TCGA cohorts, respectively (Figure 2g–i). The corresponding AUCs were 0.884, 0.830, and 0.761 for predicting 5‐year DSS (Figure 2j–l). Time‐dependent AUCs for other metrics were displayed in Figures S3 and S4.

**FIGURE 2:**
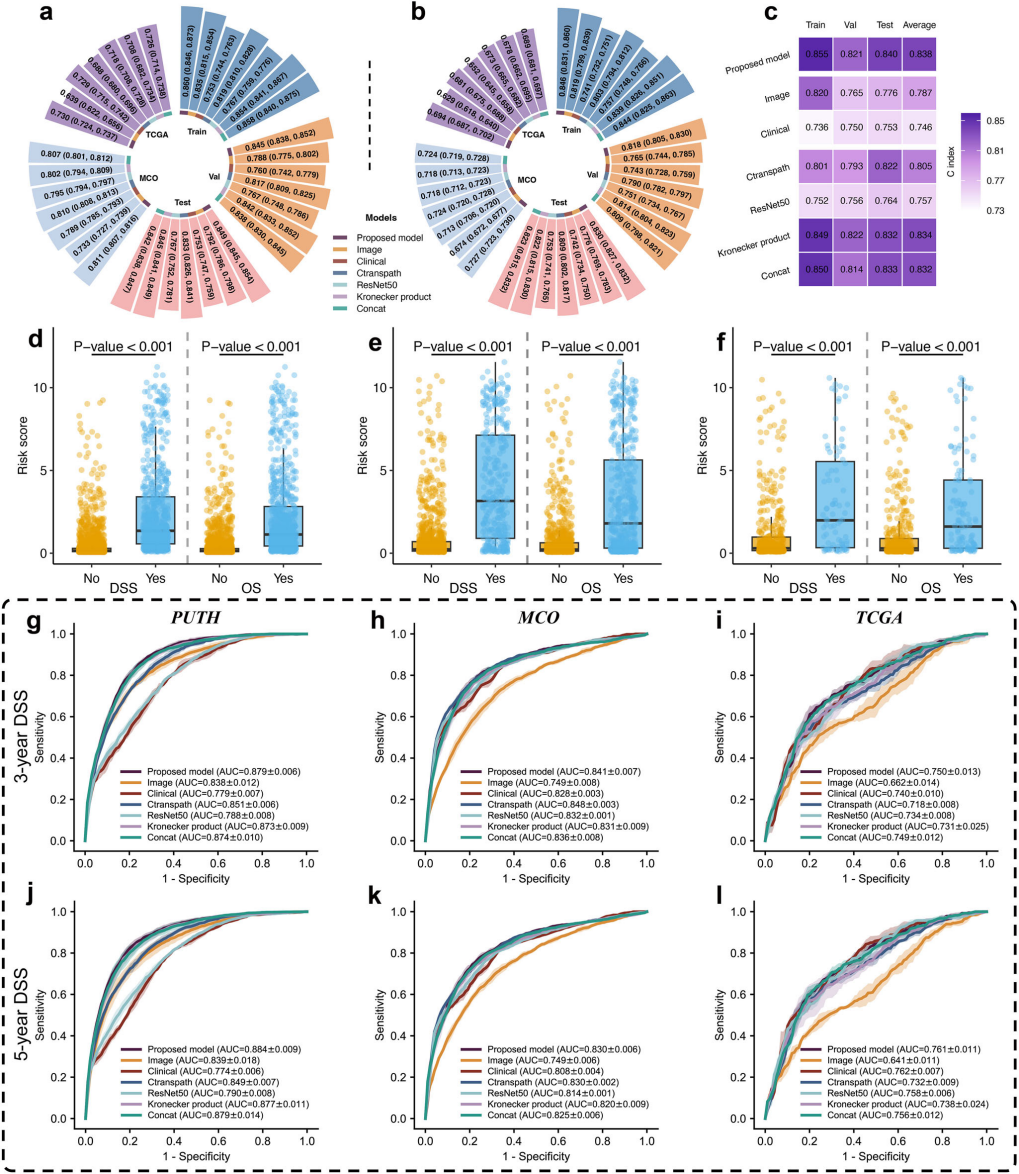
Comparative performance of prognostic models across three datasets. (a) C‐index for DSS of each model across three datasets. Mean value and 95% confidence intervals are presented. (b) C‐index for OS of each model across three datasets. (c) C‐index for DFS of each model in the PUTH cohort. (d–f) Associations between survival status and multimodal model‐derived risk scores in PUTH cohort, MCO cohort, and TCGA cohort, respectively. (g–i) ROC curves and the corresponding AUCs for 3‐year DSS of each model in the PUTH cohort, MCO cohort, and TCGA cohort, respectively. The confidence band shows AUC ± sd for the averaged ROC curves. (j–l) ROC curves and the corresponding AUCs for 5‐year DSS of each model. Abbreviations: AUC, area under the curve. DFS, disease‐free survival. DSS, disease‐specific survival. OS, overall survival. ROC, receiver operating characteristic. SD, standard deviation.

The value of our model for risk stratification was further validated through K–M analysis with the log‐rank test in both development and external datasets. By dividing the patients into two groups, significant prognostic differences were observed in the PUTH cohort, with the high‐risk group having markedly worse outcomes than the low‐risk group (Figure 3). For external validation in the MCO cohort, the multimodal model consistently demonstrated effective risk stratification (Figure 4 and Figure S5). The 5‐year DSS rates for low‐risk and high‐risk groups were 93% and 54% with a HR of 8.93 (95% CI, 6.50–12.27, P‐value < 0.001, Figure 4a,b). The corresponding 5‐year OS rates were 82% and 46% with an HR of 4.02 (95% CI, 3.27–4.93, P‐value < 0.001). The high‐risk group showed significant correlations with higher T stage, lymph node metastasis as well as AJCC stage (Figure 4c–e). Considering that clinical information was included as input for survival prediction, we further applied a multivariable Cox regression by adjusting for age, sex, and AJCC stage (Figure 4f). The analysis confirmed that deep learning model‐derived stratification was an independent prognostic factor with an adjusted HR of 3.60 (95% CI, 2.49–5.21, P‐value < 0.001) for DSS in the MCO cohort. The Cox regression for OS and that adjusting for TNM stage, was presented in Figure S6. For external validation in the TCGA cohort, the analysis results were unchanged (Figure 4g–l and Figures S5 and S6). Notably, significant survival stratification for DSS was also observed in the multivariable Cox regression, with an adjusted HR of 2.00 (95% CI, 1.05–3.80, P‐value = 0.036, Figure 4l). For patients with non‐metastatic CRC, the deep learning‐predicted high risk remained being significantly associated with poor outcomes (Figure S7). These robust results across discrepant datasets verified the reliability and generalizability of our model, highlighting its potential applicability in diverse clinical scenarios.

**FIGURE 3:**
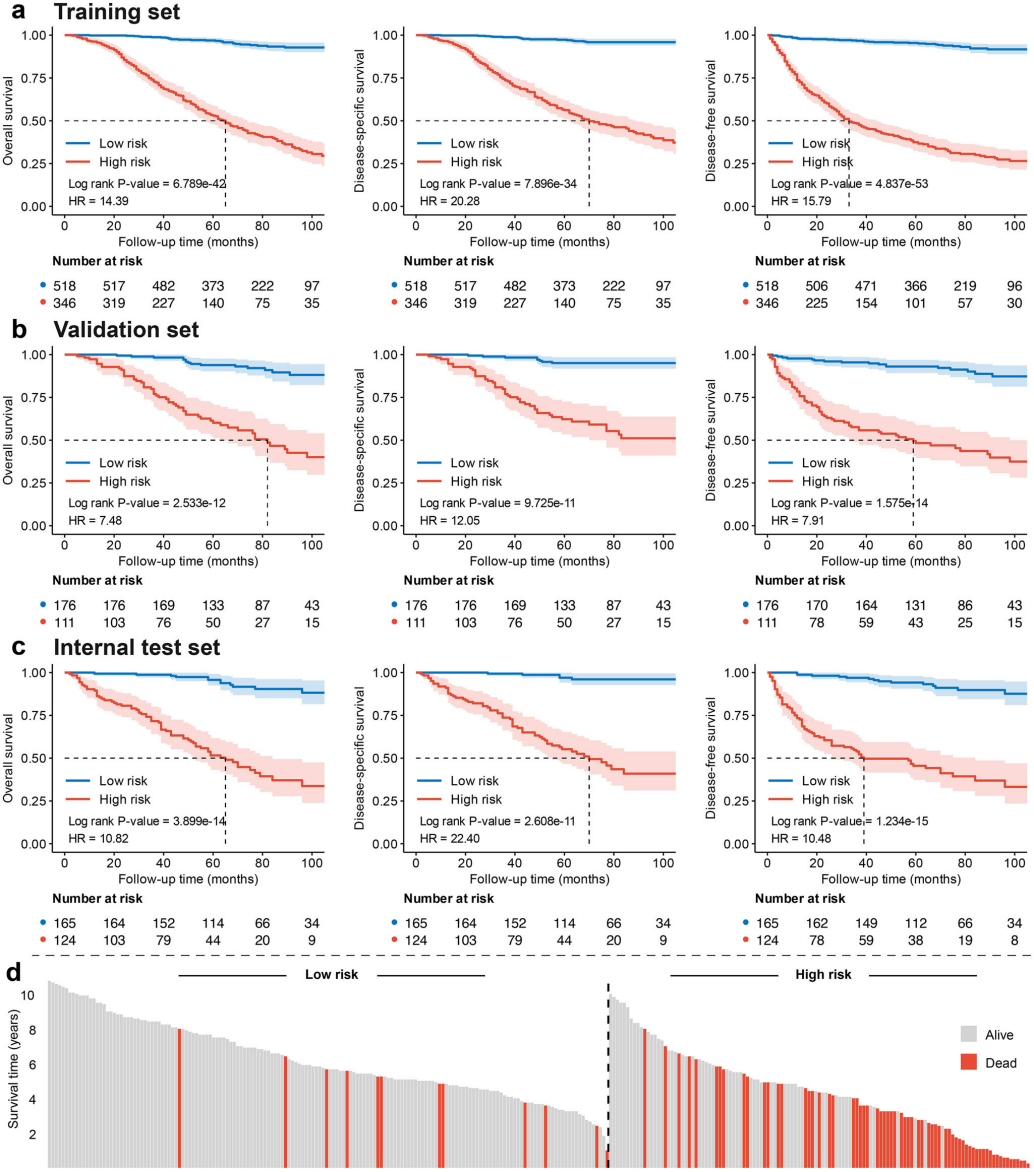
Performance evaluation of prognostic stratification in the development cohort. (a–c) Kaplan‐Meier curves for OS, DSS, and DFS using deep learning‐derived risk score in training, cross‐validation, and internal test set, respectively. The survival curves are presented with 95% confidence intervals. Patients are stratified into low‐risk and high‐risk group based on the median of prediction score. The number of patients at risk at each time point is shown at the bottom of each plot. (d) Distribution of survival time for patients in the internal test set. Abbreviations: HR, hazard ratio.

**FIGURE 4:**
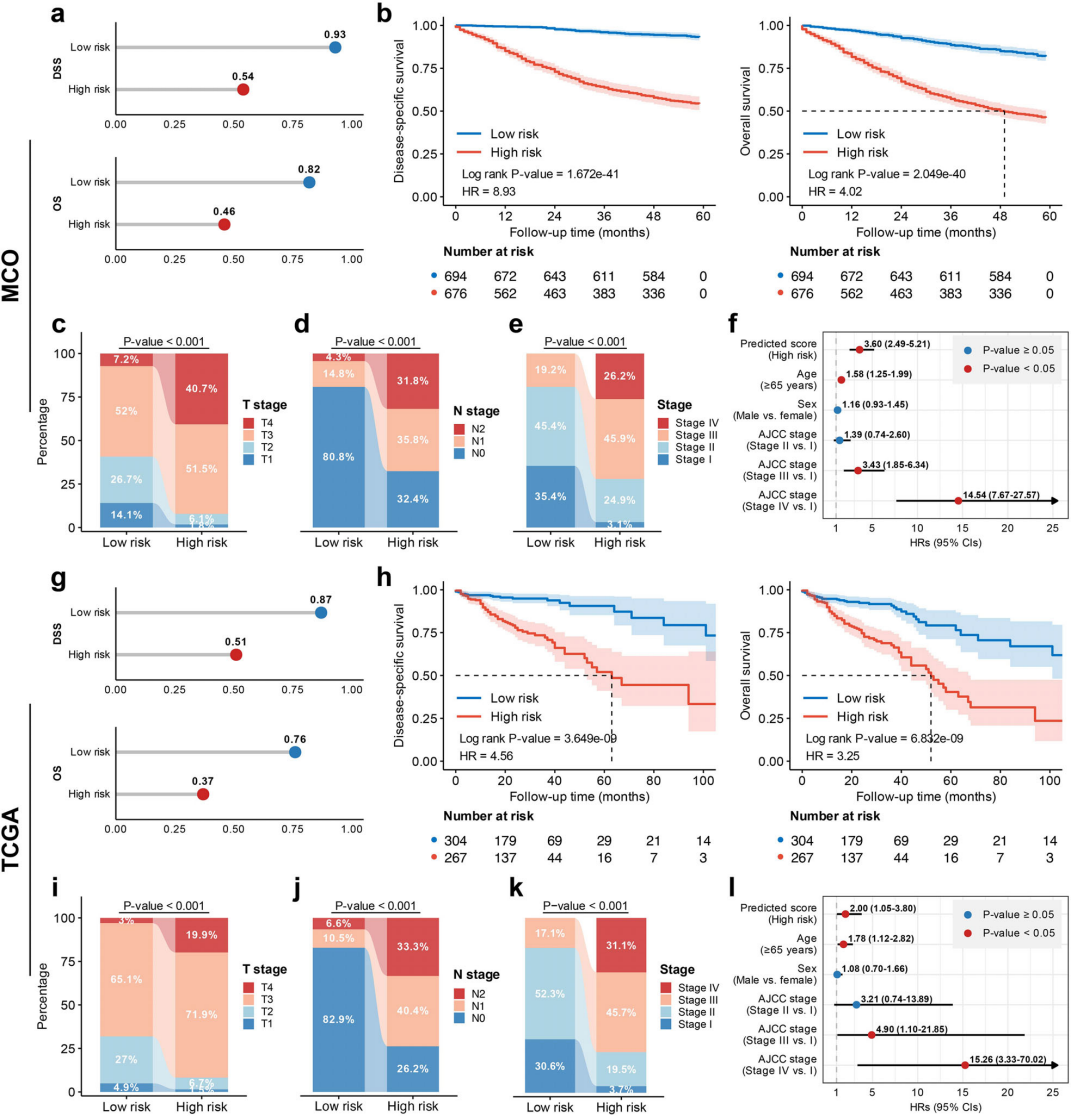
Performance evaluation of prognostic stratification in external validation cohorts. (a) The 5‐year survival rates for DSS and OS across low‐risk and high‐risk groups based on deep learning‐derived risk score in MCO cohort. (b) Kaplan–Meier curves for DSS and OS across risk groups in the MCO cohort. (c–e) The bar plots of the T stage, N stage, and AJCC stage across risk groups in the MCO cohort. P‐values are obtained by χ^2^ or Fisher's exact test. (f) Multivariate Cox regression analysis for DSS between deep learning‐predicted risk, age, sex, and AJCC stage in the MCO cohort. HRs and corresponding 95% confidence intervals are presented. (g) The 5‐year survival rates for DSS and OS across low‐risk and high‐risk groups in the TCGA cohort. (h) Kaplan‐Meier curves for DSS and OS across risk groups in the TCGA cohort. (i–k) The bar plots of the T stage, N stage, and AJCC stage across risk groups in the TCGA cohort. (l) Multivariate Cox regression analysis for DSS between deep learning‐predicted risk, age, sex, and AJCC stage in the TCGA cohort. Abbreviations: DSS, disease‐specific survival. HR, hazard ratio. OS, overall survival.

### 3.3 Subgroup Analyses and Therapeutic Benefit

We further assessed the performance of our model to recognize favorable or unfavorable patients among stage II/III CRC (Figure 5) in the MCO cohort. Figure 5a,b detailed the predicted scores and clinicopathological characteristics of the subgroup. Eventually, 168 of 483 patients with stage II CRC were identified as high‐risk, while 310 of 443 patients with stage III CRC were identified as high‐risk (Figure 5c). The correlation analyses revealed that deep learning‐derived risk was mainly related to pathological T stage (Spearman r = 0.41 in stage II and Spearman r = 0.45 in stage III), followed by the differentiation grade (Spearman r = 0.32 in stage III, Figure 5d,e). Within stage II group, the high‐risk patients exhibited a significantly worse prognosis than low‐risk patients, with a HR of 3.21 (95% CI, 1.80–5.73, P‐value < 0.001, Figure 5f). While no significant survival benefit from adjuvant chemotherapy (ACT) or from different chemotherapy regimens was observed in low‐risk group or high‐risk group of this population (Figure S8). The survival analysis in stage III CRC group showed that patients with predicted high risk had poorer prognosis (HR = 4.11, 95% CI, 2.40–7.04, P‐value < 0.001, Figure 5g). For low‐risk patients with stage III CRC, K–M analysis indicated no significant improvement in prognosis with ACT (P‐value = 0.794, Figure 5h). Conversely, ACT was significantly associated with a favorable prognostic response for high‐risk patients with stage III CRC (P‐value = 0.017, Figure 5i). The guideline‐based risk stratification was also a significant predictor of DSS for stage III patients (HR = 2.61, 95% CI, 1.80–3.79, P‐value < 0.001), while our model provided prognostic information beyond the guideline‐based risk stratification system (Figure S9). By stratifying patients into three groups combing deep learning‐predicted risk, T stage, and N stage, the re‐stratified high‐risk group had a worse prognosis in both Stage II and III (Figure S10). These results suggested that our multimodal model might provide complementary prognostic value beyond traditional TNM stage and serve as a clinically valuable tool for risk restratification and ACT decision guidance among patients with stage II/III CRC.

**FIGURE 5:**
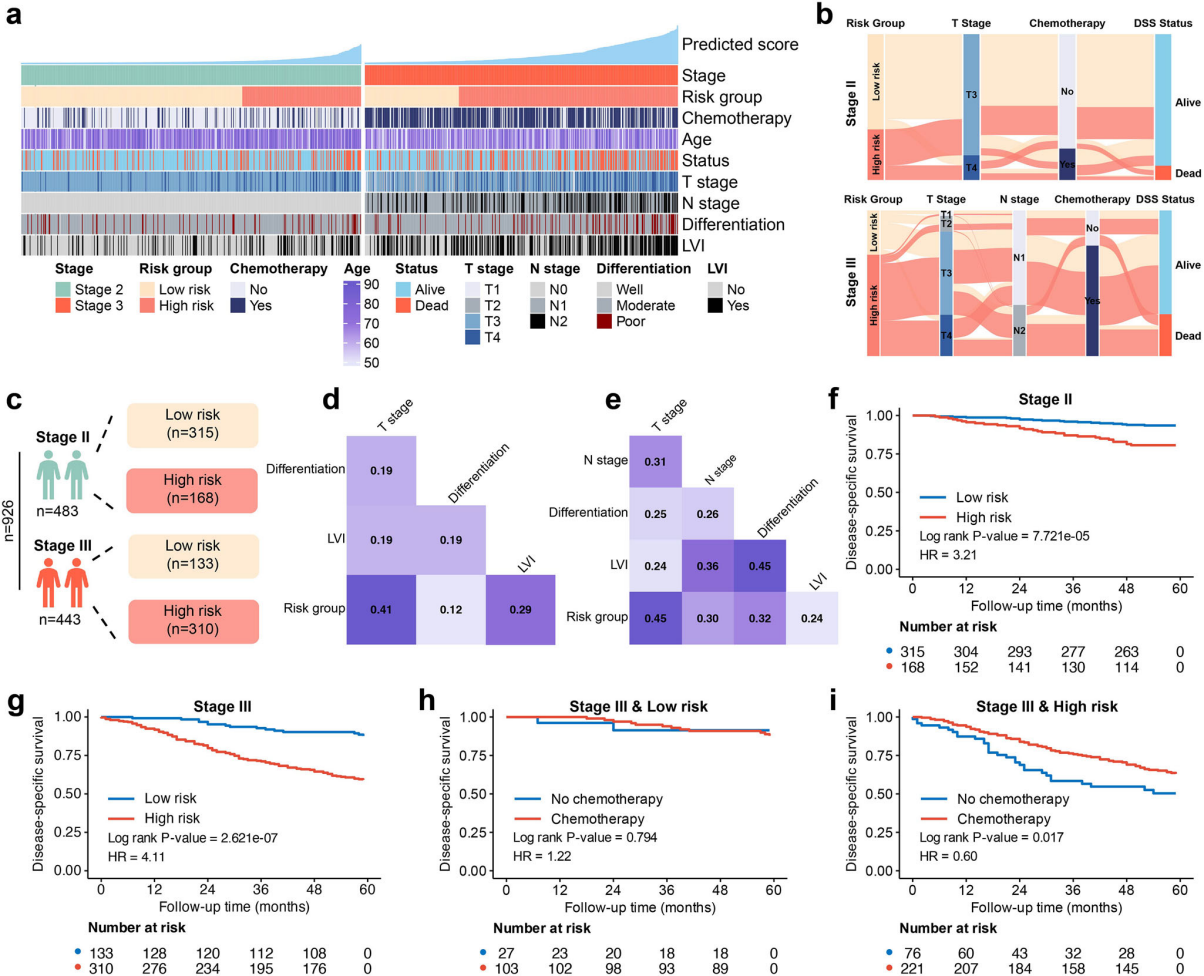
Clinicopathological characteristics and stratification effect for stage II and III colorectal cancers in MCO cohort. (a) Heatmaps of risk stratification, clinicopathological characteristics, and prognosis for patients with stage II and III colorectal cancers. (b) Alluvial diagram showing the distribution of risk stratification across different categories of T stage, N stage, adjuvant chemotherapy, and DSS status. (c) The summary of risk stratification for patients with stage II and III colorectal cancers. (d, e) Spearman correlation analyses between risk stratification and key clinicopathological risk factors for stage II (d) and III (e) colorectal cancers. (f, g) Kaplan–Meier curves for DSS across risk groups in patients with stage II (f) and III (g) colorectal cancers. (h) Kaplan–Meier curves for DSS across different chemotherapy status in low‐risk patients with stage III colorectal cancers. (i) Kaplan–Meier curves for DSS across different chemotherapy status in high‐risk patients with stage III colorectal cancers. Abbreviations: DSS, disease‐specific survival. HR, hazard ratio. LVI, lymphovascular invasion.

### 3.4 Model Interpretability and Relevance to Tumor Microenvironment

To enhance interpretability and explore the prognosis‐related patterns learned by the model, we applied heatmaps to visualize the high‐attention regions on WSI thumbnail images. Figure 6a–c and Figure S11 showed the typical cases with poor prognosis across three cohorts, where red masks indicated high attention. It could be observed that high‐attention regions were consistent with tumor epithelium in both original WSIs and magnified ROIs with cell annotations. Heatmaps of low‐risk patients were presented in Figure S12. Through confirmation from pathologists, the selected top 5 patches with highest attention scores from each high‐risk slide showed patterns of invasive growth, abundant stroma, and poorly differentiated tumor clusters (Figure 6a). To further quantitatively analyze the cell composition, cell fractions in high‐attention patches were compared across low‐risk and high‐risk groups (Figure 6d). The results showed that high‐attention areas of the model contained significantly fewer lymphocytes and necrotic cells, and more stromal cells for identifying high‐risk versus low‐risk CRC (Figure 6e and Figure S13).

**FIGURE 6:**
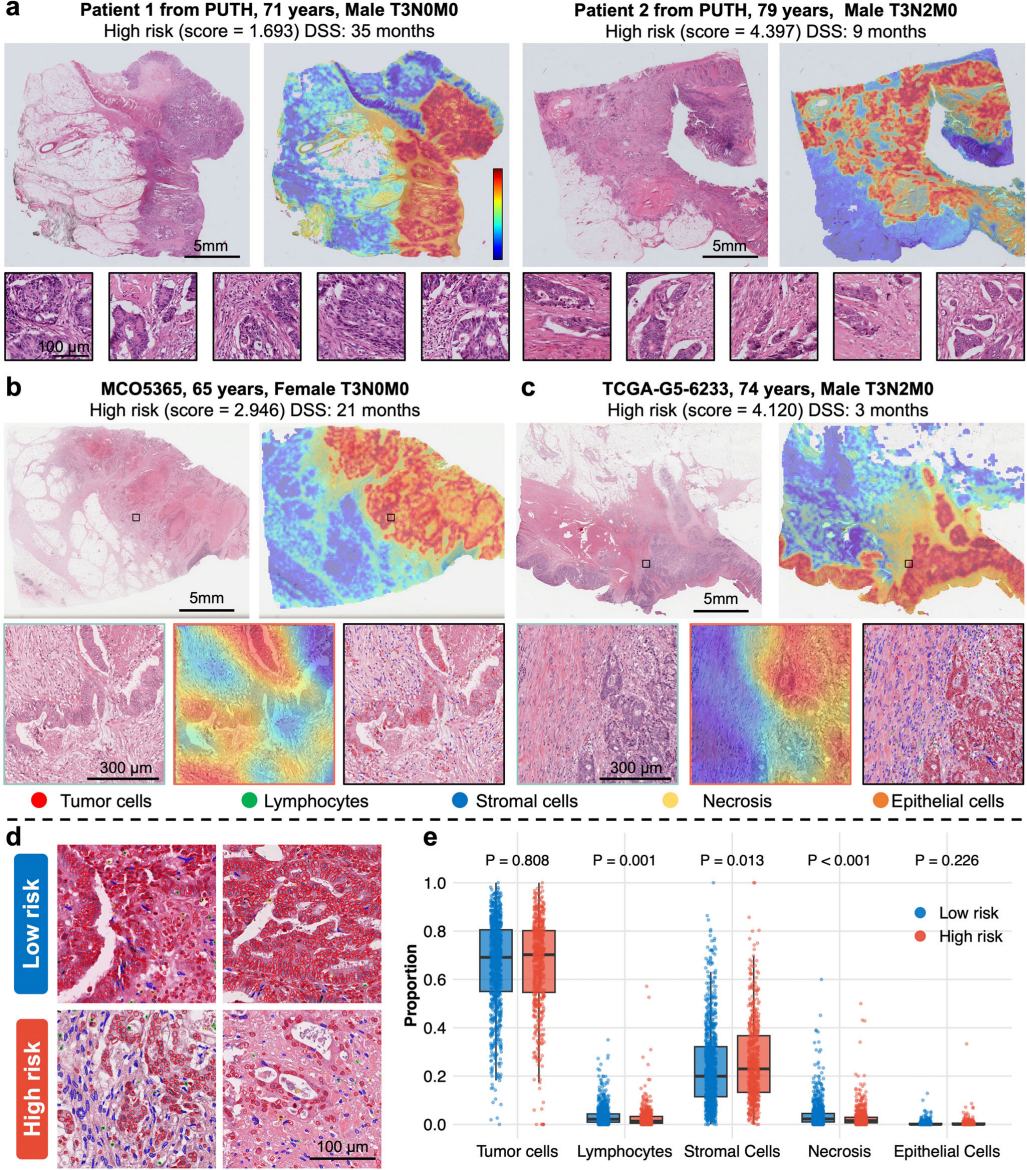
Interpretability analyses of heatmap visualization and cell quantitation. (a) Example H&E‐stained WSIs and corresponding whole‐slide attention heatmap of patients with high predicted scores from the PUTH cohort, with the red region indicating high attention intensity. The top 5 local patches with the highest attention score from each slide are shown at the bottom of each plot. Scale bar: 5 mm for slides and 100 µm for patches. (b, c) Example H&E‐stained WSIs and corresponding whole‐slide attention heatmap of patients with high predicted scores from the MCO cohort (b) and TCGA cohort (c), respectively. Fine‐grained ROIs (left), corresponding heatmaps (middle), and nuclei segmentation results (right) are shown at the bottom. Scale bar: 5 mm for slides and 300 µm for ROIs. (d) Patches with the highest attention score from low‐risk and high‐risk cases and corresponding cell annotations (in MCO cohort). Scale bar: 100 µm. (e) Quantitative comparison of cell fraction in high‐attention patches across risk groups (in MCO cohort). P‐values are obtained by t test. Abbreviations: DSS, disease‐specific survival. ROI, region of interest.

### 3.5 Model Interpretability at Molecular Level

To gain deeper insights into the intercellular spatial relationships within the tumor ecosystem across risk groups, we analyzed multi‐omic sequencing data from the PUTH cohort (Figure 7). To ensure the correspondence between molecular information and pathological information, the FFPE tissues which the histopathology images used for computational pathology analysis and the fresh tissues used for RNA sequencing were in very close proximity. 111 patients with bulk RNA sequencing data were analyzed, with 55 in the high‐risk group and 56 in the low‐risk group according to our model. 246 differential expressed genes (DEGs) were identified, with 185 unregulated and 61 downregulated genes in the high‐risk group (Figure 7a). Pathway enrichment analysis revealed that the upregulated DEGs from the high‐risk group were predominantly enriched in cytokine‐cytokine receptor interaction, ECM‐receptor interaction, vasculogenesis, and endothelial cell differentiation (Figure 7b). While pathways related to complement and coagulation cascades, immunoglobulin complex, and regulation of CD8+αβ T cell activation were significantly upregulated in the low‐risk group (Figure 7c). Immune infiltration analysis indicated that the low‐risk group had higher proportions of B cells (P‐value = 0.045) and myeloid dendritic cells (P‐value = 0.048, Figure 7d).

**FIGURE 7:**
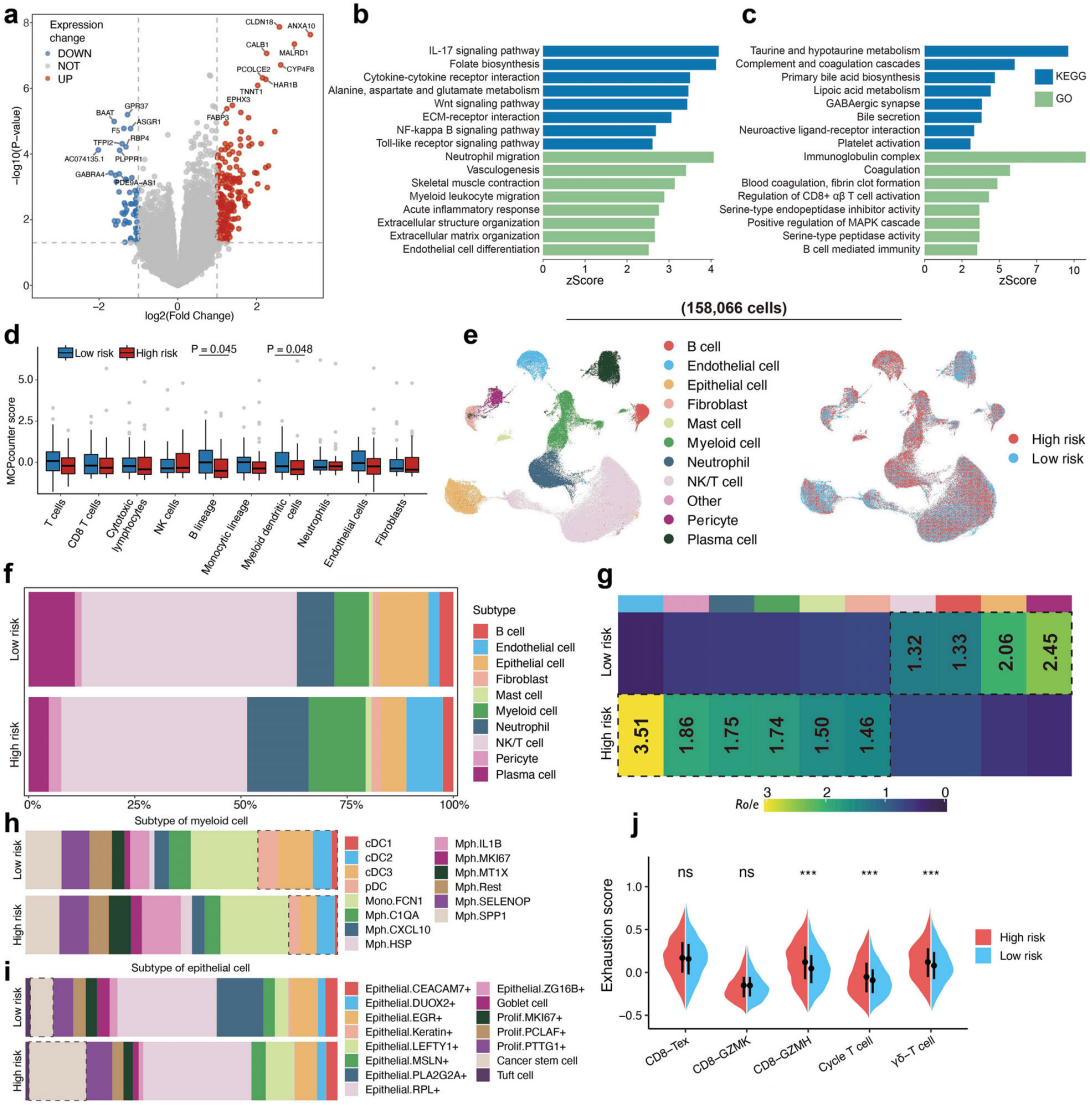
Analyses of tumor microenvironment between patients with low and high predicted risk. (a) Volcano plot displaying differentially expressed genes between the low‐risk group and high‐risk group. (b, c) Bar plot showing significantly enriched KEGG and GO pathways in the high‐risk group and low‐risk group, respectively. (d) Box plots comparing immune cell abundances in the tumor microenvironment across risk groups, as estimated by the MCPcounter algorithm. (e) UMAP dimensionality reduction of major cell types. (f) Bar plots showing the percentage of each major cell type across risk groups. (g) Tissue prevalence of each cell type estimated by Ro/e score. Ro/e denoting the ratio of observed to expected cell number. (h) Bar plots showing the percentage of each epithelial cell subset across risk groups. (i) Bar plots showing the percentage of each myeloid cell subset across risk groups. (j) Gene set scores of exhaustion among major T subsets across risk groups. Abbreviations: UMAP, Uniform Manifold Approximation and Projection.

In the 21 patients with single‐cell RNA sequencing data, 10 were in the high‐risk group, and 11 were in the low‐risk group according to our model. The pooled analysis of 158 066 cells identified 10 major cell types, with distinct transcriptional profiles between low‐ and high‐risk groups (Figure 7e and Figure S14). Notably, the low‐risk group presented an increasing trend in the abundance of plasma cells, T cells, and NK cells, which were indicative of an enhanced immune response, while the high‐risk group showed enrichment of endothelial cells, pericytes, neutrophils, and myeloid cells (Figure 7f,g), which were associated with angiogenesis and immune suppression. Then, cell sub‐clusters were identified, with higher proportions of dendritic cells (DC) in the low‐risk group, which supported the results of bulk transcriptomic analysis and indicated enhanced antigen presentation to activate adaptive immune response against the tumor (Figure 7h and Figure S15). In addition, the high‐risk group had a higher proportion of cancer stem cells (CSC) and significantly higher exhaustion scores in most T cell types (Figure 7i,j and Figure S16), indicating a tumor microenvironment of high plasticity and immunosuppression.

We further validated the aforementioned results using high‐resolution spatial transcriptomic data from the 10×Genomics Visium HD and Xenium spatial gene expression dataset portal (Figure 8), in which the gene expression and high‐attention patches could be precisely matched. For the low‐risk sample (P1CRC) according to our model, high‐attention patches showed abundant infiltration of T cells and plasma cells (Figure 8a–c), with marked enrichment of the antigen processing and presentation pathway and positive regulation of T cell activation pathway (Figure 8d). In contrast, high‐attention patches from the high‐risk sample (P5CRC) exhibited infiltration of endothelial cells and neutrophils (Figure 8e–g), with significant activation of angiogenesis and hypoxia‐inducible factor‐1 (HIF‐1) signaling pathway (Figure 8h). By extracting the top 20 high‐attention patches, the enrichment analysis revealed that adaptive immune reaction‐related pathways were significantly activated in low‐risk sample, whereas the vascular endothelial growth factor (VEGF)‐related pathways and HIF‐1 signaling pathway were significantly enriched in high‐risk sample (Figure S17). These findings demonstrated a strong association between the model‐derived scores and underlying molecular features, suggesting the interpretability of our multimodal model.

**FIGURE 8:**
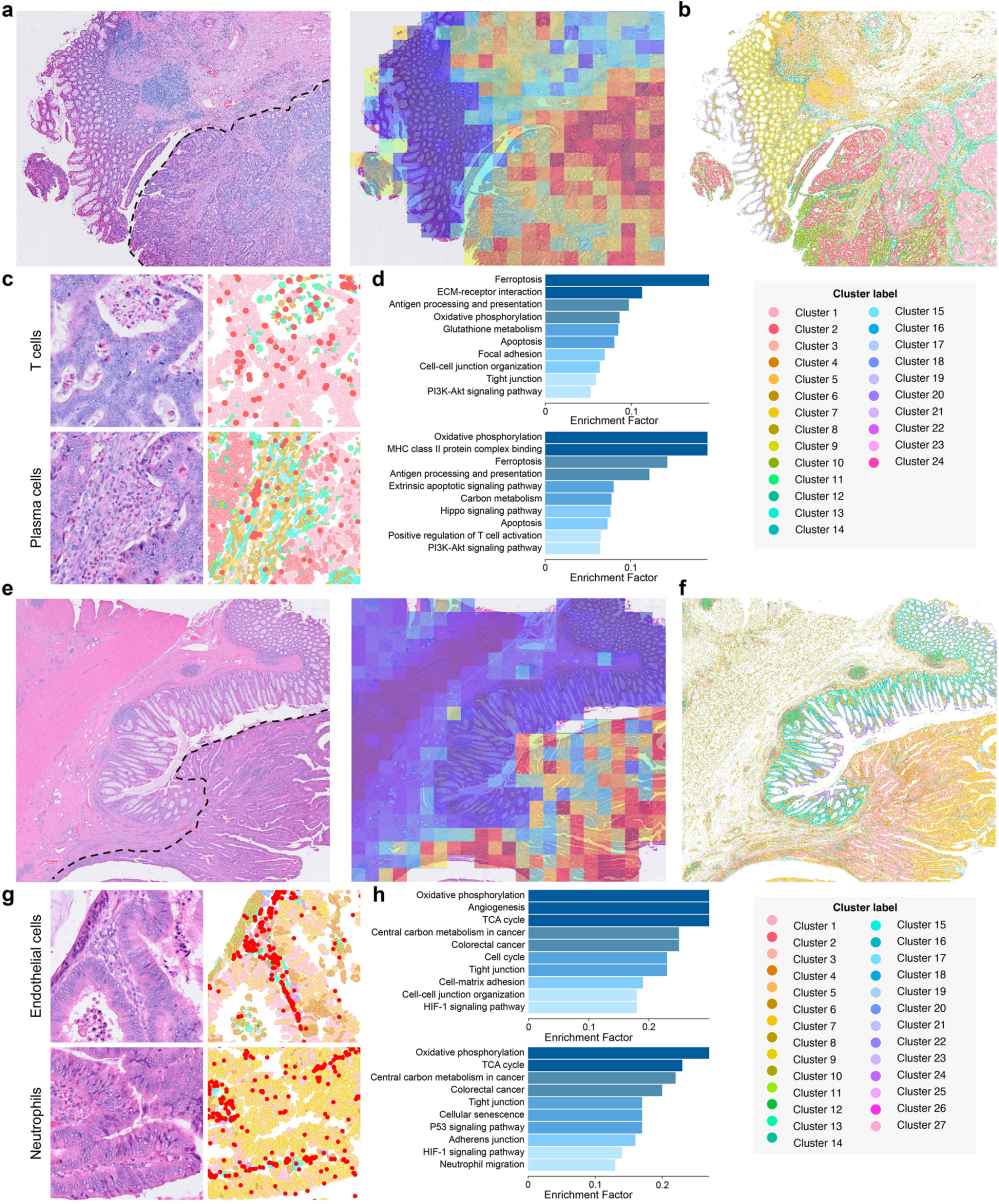
Spatial transcriptional and functional heterogeneity on high‐attention regions. (a–d) Results from a low‐risk sample (P1CRC). (e–h) Results from a high‐risk sample (P5CRC). (a, e) Visium HD H&E‐stained WSIs and corresponding whole‐slide attention heatmap. (b, f) Results from unsupervised clustering. (c, g) High‐attention patches and cell segmentation results based on spatial transcriptional profiles. T cells, plasma cells, endothelial cells, and neutrophils were annotated using red dots, respectively. (d, h) Bar plots showing significantly enriched KEGG and GO pathways in high‐attention patches.

## 4 Discussion

In this study, we developed and validated an explainable multimodal deep learning model that integrated clinical variables and H&E‐stained histopathological WSIs for accurate prognosis prediction in CRC patients following radical surgery. The proposed model demonstrated consistently superior predictive performance through external validation across large cohorts from independent medical centers, underscoring its robustness and reliable predictive capability. Moreover, the model maintained interpretability by capturing well‐established histopathological features related to worse outcomes. Importantly, a strong association between the model‐derived scores and underlying molecular features was confirmed by comprehensive multi‐omic analyses, suggesting its potential as a practical auxiliary tool for guiding individualized treatment strategies for CRC patients. To the best of our knowledge, it is the first study to combine a computational pathology model with multi‐omic sequencing data, including bulk transcriptome, single‐cell transcriptome, and high‐resolution spatial transcriptome.

Accurate prediction of prognosis is integral to the management of CRC in clinic since risk stratification influenced the treatment decisions and follow‐up strategies after surgical resection [28]. Nevertheless, it remains a significant challenge due to extensive tumor heterogeneity and complex molecular landscape [29, 30]. Recent studies have reported exceptional predictive performance of histopathology‐based deep learning models, while most of these researches predominantly applies unimodal architectures, and incorporating clinical variables remains unexplored [11, 12]. Our approach employs cross‐attention mechanism for comprehensive integration of pathological features and clinical features, which achieves substantial prediction improvements compared to single‐modality models and also outperforms traditional multimodal methods. Notably, we use UNI2, a pathology foundation model based on the DINOv2 backbone, for patch‐level feature extraction, which exhibited enhanced performance across datasets, reinforcing that strong and meaningful image representations enable downstream tasks in histopathology workflows [23]. Additionally, in the multivariate Cox regression analysis that adjusts for clinicopathological factors, the risk score derived from our multimodal model consistently shows significant relevance to survival outcomes, highlighting it as an independent predictor of poor prognosis.

Subgroup analyses suggest that our model can identify patients who would benefit most from postoperative ACT. Among patients with stage III CRC for whom adjuvant therapy is the standard treatment, ACT was associated with significantly improved survival in high‐risk patients but not in low‐risk patients (Figure 5). The discrepant chemotherapy benefits observed between risk groups have potential clinical implications. The relatively low recurrence risk observed in the low‐risk group reinforces that these patients face a fundamentally favorable prognosis, suggesting that de‐escalated treatment may be prescribed, especially for patients with low treatment willingness, advanced age, or significant comorbidities. High‐risk patients embody more aggressive and proliferative tumor biology, therefore, more intensive treatment would be applicable for this group [31, 32]. Although patients with stage III CRC can be categorized into low‐risk and high‐risk groups based on T and N stage [31], our deep‐learning model further stratifies the clinically defined low‐risk (T1‐3, N1) group into low‐ and high‐risk subgroups and similarly refines the clinically defined high‐risk (T4 or N2) group (Figure S9b,c), demonstrating that our model complements current clinical risk factors. Among patients with stage II CRC, subgroup analyses reveal that our model successfully identifies a substantial portion of low‐risk patients with excellent outcomes. The further three‐group reclassification by incorporating the deep learning score and pathological T stage achieves better separation, with 5‐year DSS rates of approximately 94%, 89%, and 63% in low‐, medium‐, and high‐risk groups, respectively (Figure S10). The findings suggest that these low‐risk stage II patients might avoid adjuvant treatment and associated toxicities for the favorable prognosis [33, 34]. However, the risk stratification for stage II CRC doesn't predict benefit from chemotherapy in this retrospective cohort, which requires further prospective study to definitively validate the impact of ACT within each risk group.

The investigation of explainability provides good prospects to enhance the understanding into the decision‐making procession of our model. By visualizing the contributions of different regions on WSIs, the results confirm that important areas closely correspond to tumor regions, which empirically reflect the degree of malignancy, in both low‐risk and high‐risk patients. Specifically, characteristic morphological patterns related to a worse prognosis are displayed on high‐attention patches from high‐risk patients, including poorly differentiated tumor clusters and desmoplastic stroma reaction [35, 36]. While patches from favorable patients show well‐formed glandular duct structures and lower nuclear pleomorphism of tumor cells. Further cell quantitation and multi‐omic analyses reveal the distinct microenvironment of more lymphocyte and DC infiltration in low‐risk patients, indicating a stronger anti‐tumor adaptive immunity, which restrains the progression of tumor [37, 38]. On the other hand, in the high‐risk group, endothelial cells, pericytes and neutrophils are enriched, while the major T cell types are significantly exhausted, indicating enhanced angiogenesis and suppressed anti‐tumor immune response [39]. Noteworthily, the high‐resolution spatial transcriptomic analysis is utilized to elucidate the functional heterogeneity in high‐attention patches. Unfavorable pathways are activated in patches from the high‐risk sample, such as vasculogenesis‐related and HIF‐1 signaling pathway, reflecting a hostile microenvironment characterized by hypoxia [40]. These results provide evidence of interpretability that our model can harnesses biology‐relevant features of tumor microenvironment for prognosis prediction and might facilitate AI‐powered pathological biomarker discovery.

The proposed multimodal model holds considerable promise for practical implementation because it requires only histopathological images and broadly available clinical variables. However, the study has several limitations. First, the model was trained and evaluated on retrospective datasets, despite validation on external cohorts, potential selection bias and confounding variables might not be eliminated. Second, heterogeneity across institutions challenged the model's generalizability. Although the employment of a pathology foundation model reduced the bias introduced by sample preparation [23], differences in ethnicity, treatment patterns, and follow‐up still persisted. Specifically, there was disparity in the frequency of driver gene variants by race, which mediated the heterogeneity of tumor biology and treatment response, and the model may not fully capture these effects especially when it was applied on cross‐national cohorts [41, 42]. On the other hand, the follow‐up period of the TCGA cohort was relatively shorter compared to PUTH and MCO cohorts, which hindered the determination of outcome events and limited the statistical power. While prediction performance of our model decreased slightly in the TCGA cohort, the risk stratification remained a risk factor independent of the AJCC stage, demonstrating the superior generalizability. Third, as an observational study, the therapeutic intervention to directly evaluate treatment strategies was not involved. The observed associations between risk stratification and benefit from ACT should be interpreted with caution. Finally, multi‐omics data were not integrated for the model development. Integrative multi‐omics analyses enhanced the prognostic performance, however, obtaining massive molecular data remained costly and time‐consuming [43, 44]. Recent studies showed that deep leaning could be applied to predict gene expression from routine histopathology slides, emerging as a novel and promising pattern to generate complementary transcriptomic information for prognosis prediction [45, 46, 47]. While the prediction accuracy of current models varies substantially, which could not yet substitute for molecular testing and warrant further exploration [48]. Future studies should focus on the incorporation of clinical variables, histopathological slides, and multi‐omics data to attain more precise prediction and conduct prospective validations in real‐world scenarios.

## 5 Conclusions

In conclusion, we developed and validated an explainable multimodal fusion model integrating key clinical variables and histopathological images, which demonstrated superior accuracy and generalizability for predicting survival outcomes in CRC. Our model achieves substantial improvements in predictive performance, enabling more precise risk stratification and potentially optimizing individualized treatment decisions. These findings make it possible to serve as a complementary and cost‐efficient auxiliary tool for the management of CRC in clinical workflow.

## Author Contributions

Z.J., W.F., and X.Z. conceived the study. Z.J. and C.S. did the data collection and processing. Z.J., X.S., and Y.M. conducted experiments and data analysis and wrote the manuscript. Z.J., X.S., C.S., Y.M., R.L., M.C., J.C., N.H., R.W., X.B., J.J., W.F., and X.Z. contributed materials, methodology, and clinical expertise. W.F. and X.Z. supervised the work. All authors contributed to the experimental design, the interpretation of the results, and editing of the final manuscript. All authors had full access to the data, and J.Z., X.S., J.J., W.F., and X.Z. directly accessed and verified the data in the study. All authors accept the final responsibility to submit for publication and take responsibility for the contents of the manuscript.

## Ethics Statement

Ethical approval was obtained from the Institutional Review Boards of Peking University Third Hospital (IRB00006761‐M2024534), and this study adhered to the tenets of the Declaration of Helsinki. The need for informed consent was waived by the Institutional Review Boards of Peking University Third Hospital.

## Consent

Patients and/or the public were not involved in the design, conduct, reporting or dissemination plans of this research.

## Conflicts of Interest

The authors declare no conflicts of interest.

## Supporting information




**Supporting File 1**: advs78417‐sup‐0001‐SuppMat.docx

## Data Availability

The MCO dataset is available upon submission and approval of a data access request through the following portal: http://www.sredhconsortium.org/sredh‐datasets/mco‐study‐whole‐slide‐image‐dataset. The TCGA data (clinicopathological information and digital slides) used in this study are available at https://portal.gdc.cancer.gov/. The spatial transcriptomic data from 10×Genomics Visium HD and Xenium spatial gene expression dataset portal are available at https://www.10xgenomics.com/products/visium‐hd‐spatial‐gene‐expression/dataset‐human‐crc. The remained data that support the findings of this study are available from the corresponding author (Xin Zhou, zhouxinasd@sina.cn) upon reasonable request.
