# Supplementary material for: An Explainable Multimodal Deep‐Learning Model Using Histopathological Image and Clinical Information for Prognosis Prediction in Colorectal Cancer: A Multi‐Center Cohort Study
Source: Adv Sci (Weinh). 2026 Oct 26:e78417. Online ahead of print. doi: 10.1002/advs.78417 (PMC13710700; doi:10.1002/advs.78417)
Supplement: Supplementary file 1 [file ADVS-9999-e78417-s001.docx]

**An explainable multimodal deep-learning model using histopathological image and clinical information for prognosis prediction in colorectal cancer: a multi-center cohort study**

**Supplementary**

**Tables**

[Supplementary Table S1. Details about patient enrollment and slide scanning.	4](#_Toc237470230)

[Supplementary Table S2. Implementation details of the model.	5](#_Toc237470231)

[Supplementary Table S3. Characteristics of CRC patients for single-cell RNA sequencing.	6](#_Toc237470232)

[Supplementary Table S4. Markers for identification of major cell types.	7](#_Toc237470233)

[Supplementary Table S4. Clinical characteristics of CRC patients in training, validation, internal test sets.	8](#_Toc237470234)

[Supplementary Table S5. C-indexes of DSS and OS for stage I–III patients in each cohort.	9](#_Toc237470235)

**Figures**

[Supplementary Fig.S1: Flowchart of patient inclusion and exclusion.	10](#_Toc237470236)

[Supplementary Fig.S3: ROC curves and the corresponding AUCs for 3-year and 5-year OS.	12](#_Toc237470237)

[Supplementary Fig.S4: ROC curves and the corresponding AUCs for 3-year and 5-year DFS.	13](#_Toc237470238)

[Supplementary Fig.S5: Distribution of survival time for patients in the external sets.	14](#_Toc237470239)

[Supplementary Fig.S6: Multivariate Cox regression analyses between deep learning-predicted risk and clinicopathological variables.	15](#_Toc237470241)

[Supplementary Fig.S7: Multivariate Cox regression analyses between deep learning-predicted risk and clinicopathological variables in patients with non-metastatic colorectal cancer.	16](#_Toc237470242)

[Supplementary Fig.S8: Kaplan–Meier curves for DSS across different chemotherapy status or chemotherapy regimens in patients with stage II colorectal cancers.	17](#_Toc237470243)

[Supplementary Fig.S9: Guideline-based risk restratification of patients with stage III colorectal cancer in the MCO cohort.	18](#_Toc237470244)

[Supplementary Fig.S10: Integrating deep learning-derived risk with T stage and N stage for risk restratification of patients with stage II and III colorectal cancers in the MCO cohort.	19](#_Toc237470245)

[Supplementary Fig.S11: Heatmap visualization of patients with high predicted scores from PUTH cohort, MCO cohort, and TCGA cohort, respectively.	20](#_Toc237470246)

[Supplementary Fig.S12: Heatmap visualization of patients with low predicted scores from PUTH cohort, MCO cohort, and TCGA cohort, respectively.	21](#_Toc237470247)

[Supplementary Fig.S13: Interpretability analyses of quantitation in TCGA cohort.	22](#_Toc237470248)

[Supplementary Fig.S14: Expression of canonical marker genes for each major cell type.	23](#_Toc237470249)

[Supplementary Fig.S15: Dimensionality reduction for myeloid cells and epithelials.	24](#_Toc237470250)

[Supplementary Fig.S16: Dimensionality reduction and percentage of each subset for T cells.	25](#_Toc237470251)

[Supplementary Fig.S17: Enrichment analysis among top 20 patches between the low-risk and high-risk sample.	26](#_Toc237470252)

Supplementary Table S1. Details about patient enrollment and slide scanning.

| **Cohort** | **Patient (n=3381)** | **Slide (n=5182)** | **Scanner** | **Magnification** | **Format** |
| --- | --- | --- | --- | --- | --- |
| PUTH 1  (For model training, cross-validation, and internal test) | 1440 patients (from China) who underwent radical surgical resection for colorectal adenocarcinoma between January 2012 and June 2023 | 3171 slides.  Two senior pathologists independently reviewed the H&E-stained slides and selected regions from the most representative areas of the tumors | NanoZoomer S360 scanner C13220-01 (Hamamatsu, Japan) | 20× or 40× | .ndpi |
| PUTH 2  (For bulk RNA sequencing) | 111 patients who underwent radical surgical resection for colorectal adenocarcinoma between 2024 and 2025 | 111 slides | NanoZoomer S360 scanner C13220-01 (Hamamatsu, Japan) | 20× | .ndpi |
| PUTH 3  (For single-cell RNA sequencing) | 21 patients who underwent radical surgical resection for colorectal adenocarcinoma between 2023 and 2024 | 21 slides | NanoZoomer S360 scanner C13220-01 (Hamamatsu, Japan) | 20× | .ndpi |
| MCO  (For external validation) | 1370 patients (from Australia, ttps://researchdata.edu.au/mco-study-tumour-collection/1957427) | 1440 slides | Leica Aperio scanner | 40× | .svs |
| TCGA  (For external validation) | 571 patients (from USA, https://portal.gdc.cancer.gov/) | 571 slides | Leica Aperio scanner | 40× | .svs |

Supplementary Table S2. Implementation details of the model.

| **Module** | **Parameter** | **Setting** |
| --- | --- | --- |
| Overall | Batch size | 1 |
|  | Epoch | 10 |
|  | Optimizer | Adam |
|  | Learning rate | 1×10^−5^ |
|  | Weight decay | 1×10^−5^ |
|  | Time bins for survival prediction | 4 |
| Clinical encoder | Hidden dimension | 128 |
| Patch encoder | Feature dimension | 1536 |
| Attention-based MIL | Hidden dimension | 512 |
|  | Droupout | 0.25 |
| Cross-attention module | Number of head | 1 |
|  | Attention dimension | 512 |
|  | Droupout | 0.25 |

All models were trained using a single NVIDIA RTX 4090 24GB GPU on a Linux operating system. The models were trained 10 epochs and the best model checkpoint with the highest C-index on cross-validation was saved and then validated on internal test and external test datasets.

The proposed model was compared with unimodal models and models using other pretrained encoders (ResNet50[1] and Ctranspath[2]) or multimodal fusion methods (feature concatenation and PORPOISE[3]). These baseline methods were trained with the same data splits and parameters to ensure fair comparisons.

**Reference:**

1.	He K, Zhang X, Ren S, et al. Deep Residual Learning for Image Recognition. 2016 IEEE Conference on Computer Vision and Pattern Recognition (CVPR). 2015:770-8.

2.	Wang X, Yang S, Zhang J, et al. Transformer-based unsupervised contrastive learning for histopathological image classification. Med Image Anal. 2022;81:102559.

3.	Chen RJ, Lu MY, Williamson DFK, et al. Pan-cancer integrative histology-genomic analysis via multimodal deep learning. Cancer Cell. 2022;40(8):865-78.e6.

Supplementary Table S3. Characteristics of CRC patients for single-cell RNA sequencing.

| **Group** | **Patient id** | **Grade** | **Location** | **Age** | **Sex** | **AJCC stage** |
| --- | --- | --- | --- | --- | --- | --- |
| Low risk | SC01 | Moderately | Left | 55 | Male | 3 |
| High risk | SC02 | Moderately to poorly | Left | 69 | Male | 3 |
| Low risk | SC03 | Moderately | Right | 64 | Male | 3 |
| Low risk | SC04 | Poorly | Right | 72 | Male | 2 |
| Low risk | SC05 | Mucinous | Right | 68 | Female | 2 |
| Low risk | SC06 | Moderately | Sigmoid | 70 | Female | 2 |
| High risk | SC07 | Moderately | Left | 70 | Male | 4 |
| High risk | SC08 | Mucinous | Sigmoid | 80 | Female | 3 |
| High risk | SC09 | Moderately | Rectosigmoid | 60 | Female | 3 |
| Low risk | SC10 | Moderately | Right | 84 | Male | 2 |
| Low risk | SC11 | Mucinous | Rectum | 59 | Female | 3 |
| High risk | SC12 | Moderately | Rectum | 58 | Female | 2 |
| Low risk | SC13 | Moderately | Right | 71 | Female | 1 |
| High risk | SC14 | Moderately | Rectosigmoid | 50 | Male | 3 |
| Low risk | SC15 | Moderately | Sigmoid | 55 | Female | 2 |
| High risk | SC16 | Moderately | Sigmoid | 58 | Male | 3 |
| Low risk | SC17 | Moderately | Right | 70 | Female | 2 |
| High risk | SC18 | Moderately | Sigmoid | 70 | Female | 4 |
| High risk | SC19 | Moderately | Right | 59 | Female | 4 |
| Low risk | SC20 | Moderately | Sigmoid | 60 | Female | 2 |
| High risk | SC21 | Moderately | Right | 86 | Female | 3 |

Supplementary Table S4. Markers for identification of major cell types.

| **Cell type** | **Marker** |
| --- | --- |
| Epithelial cells | EPCAM, KRT18 |
| B cells | CD79A, MS4A1 |
| Fibroblast | COL3A1, COL1A1 |
| Endothelial cells | PECAM1, VWF |
| Mast cells | KIT, TPSAB1 |
| Myeloid cells | CD68, LYZ |
| Neutrophils | CSF3R, S100A9, S100A8 |
| Natural killer (NK)/T cells | CO3D, CD3E |
| Pericytes | RGS5, PDGFRB |
| Plasma cells | MZB1 |

Supplementary Table S5. Clinical characteristics of CRC patients in training, validation, internal test sets.

| **Characteristic** | **Train**  **(N = 864)** | **Validation**  **(N = 287)** | **Test**  **(N = 289)** | **P-value** |
| --- | --- | --- | --- | --- |
| **Gender** |  |  |  | 0.707 |
| male | 503 (58%) | 175 (61%) | 169 (58%) |  |
| female | 361 (42%) | 112 (39%) | 120 (42%) |  |
| **Age (years)** | 66 [58, 75] | 65 [58, 72] | 67 [58, 75] | 0.633 |
| **Tumor location** |  |  |  | 0.052 |
| left | 322 (37%) | 117 (41%) | 94 (33%) |  |
| right | 237 (27%) | 76 (26%) | 103 (36%) |  |
| rectum | 305 (35%) | 94 (33%) | 92 (32%) |  |
| **T stage** |  |  |  | 0.225 |
| T1 | 86 (10%) | 24 (8%) | 38 (13%) |  |
| T2 | 183 (21%) | 64 (22%) | 61 (21%) |  |
| T3 | 497 (58%) | 162 (56%) | 146 (51%) |  |
| T4 | 98 (11%) | 37 (13%) | 44 (15%) |  |
| **N stage** |  |  |  | 0.157 |
| N0 | 538 (62%) | 180 (63%) | 182 (63%) |  |
| N1 | 228 (26%) | 88 (31%) | 81 (28%) |  |
| N2 | 98 (11%) | 19 (7%) | 26 (9%) |  |
| **M stage** |  |  |  | 0.978 |
| M0 | 818 (95%) | 271 (94%) | 274 (95%) |  |
| M1 | 46 (5%) | 16 (6%) | 15 (5%) |  |
| **AJCC stage** |  |  |  | 0.999 |
| Stage I | 224 (26%) | 75 (26%) | 75 (26%) |  |
| Stage II | 303 (35%) | 100 (35%) | 102 (35%) |  |
| Stage III | 291 (34%) | 96 (33%) | 97 (34%) |  |
| Stage IV | 46 (5%) | 16 (6%) | 15 (5%) |  |
| **Follow-up duration (months)** | 67 [47, 91] | 69 [50, 91] | 61 [44, 84] | 0.069 |
| **OS status** | 226 (26%) | 68 (24%) | 79 (27%) | 0.586 |
| **DSS status** | 190 (22%) | 53 (18%) | 65 (22%) | 0.396 |
| **DFS status** | 270 (31%) | 80 (28%) | 86 (30%) | 0.546 |

Data are presented as median (IQR) for continuous variables and number (%) for categorial variables.

Supplementary Table S6. C-indexes of DSS and OS for stage I–III patients in each cohort.

| **DSS** | **Methods** | **PUTH**  **Training** | **PUTH**  **Validation** | **PUTH**  **Test** | **MCO** | **TCGA** |
| --- | --- | --- | --- | --- | --- | --- |
|  | Image | 0.836 (0.812, 0.860) | 0.798 (0.787, 0.810) | 0.808 (0.802, 0.814) | 0.712 (0.705, 0.720) | 0.620 (0.603, 0.637) |
|  | Clinical | 0.711 (0.698, 0.725) | 0.711 (0.691, 0.731) | 0.733 (0.726, 0.740) | 0.708 (0.701, 0.716) | 0.657 (0.636, 0.679) |
|  | ResNet50 | 0.731 (0.723, 0.738) | 0.720 (0.696, 0.745) | 0.749 (0.731, 0.767) | 0.722 (0.719, 0.725) | 0.643 (0.625, 0.660) |
|  | Ctranspath | 0.800 (0.791, 0.810) | 0.789 (0.773, 0.805) | 0.832 (0.823, 0.841) | 0.745 (0.742, 0.749) | 0.593 (0.582, 0.604) |
|  | Concat | 0.847 (0.826, 0.869) | 0.821 (0.814, 0.828) | 0.841 (0.835, 0.847) | 0.746 (0.740, 0.751) | 0.654 (0.632, 0.676) |
|  | Kronecker product | 0.844 (0.829, 0.858) | 0.825 (0.811, 0.838) | 0.845 (0.841, 0.849) | 0.737 (0.728, 0.746) | 0.624 (0.592, 0.657) |
|  | ***Proposed model*** | **0.850 (0.835, 0.866)** | **0.828 (0.818, 0.839)** | **0.851 (0.845, 0.856)** | **0.750 (0.745, 0.756)** | **0.668 (0.654, 0.682)** |
| **OS** | Image | 0.817 (0.792, 0.842) | 0.768 (0.751, 0.785) | 0.787 (0.780, 0.795) | 0.640 (0.636, 0.644) | 0.612 (0.603, 0.621) |
|  | Clinical | 0.703 (0.689, 0.716) | 0.698 (0.681, 0.715) | 0.724 (0.715, 0.733) | 0.640 (0.629, 0.651) | 0.643 (0.632, 0.653) |
|  | ResNet50 | 0.723 (0.716, 0.730) | 0.708 (0.687, 0.728) | 0.737 (0.722, 0.752) | 0.649 (0.641, 0.656) | 0.627 (0.614, 0.640) |
|  | Ctranspath | 0.783 (0.776, 0.791) | 0.759 (0.744, 0.774) | 0.806 (0.797, 0.815) | 0.657 (0.651, 0.663) | 0.593 (0.583, 0.604) |
|  | Concat | 0.832 (0.810, 0.855) | 0.789 (0.777, 0.800) | 0.820 (0.810, 0.831) | 0.659 (0.655, 0.664) | 0.643 (0.630, 0.655) |
|  | Kronecker product | 0.827 (0.813, 0.840) | 0.792 (0.783, 0.801) | 0.819 (0.811, 0.828) | 0.650 (0.646, 0.654) | 0.625 (0.605, 0.645) |
|  | ***Proposed model*** | **0.835 (0.818, 0.852)** | **0.798 (0.783, 0.812)** | **0.829 (0.826, 0.832)** | **0.662 (0.658, 0.665)** | **0.647 (0.635, 0.660)** |

The best and second-best scores were presented in bold and underline, respectively. Abbreviations: DSS, disease-specific survival. OS, overall survival. MCO, Molecular and Cellular Oncology. PUTH, Peking University Third Hospital. TCGA, The Cancer Genome Atlas.

Supplementary Fig.S1: Flowchart of patient inclusion and exclusion. a) PUTH cohort. b) MCO cohort. c) TCGA dataset. Abbreviations: MCO, Molecular and Cellular Oncology. PUTH, Peking University Third Hospital. TCGA, The Cancer Genome Atlas.

 Supplementary Fig.S2: The distribution of predicted scores from each model across three datasets. a) The distribution of predicted scores from each model in PUTH cohort. b) The distribution of predicted scores from each model in MCO cohort. c) The distribution of predicted scores from each model in TCGA cohort. Abbreviations: MCO, Molecular and Cellular Oncology. PUTH, Peking University Third Hospital. TCGA, The Cancer Genome Atlas.

Supplementary Fig.S3: ROC curves and the corresponding AUCs for 3-year and 5-year OS. a-c) ROC curves and the corresponding AUCs for 3-year OS of each model in PUTH cohort, MCO cohort, and TCGA cohort, respectively. The confidence band shows AUC ± sd for the averaged ROC curves. d-f) ROC curves and the corresponding AUCs for 5-year OS of each model. Abbreviations: OS, overall survival. MCO, Molecular and Cellular Oncology. PUTH, Peking University Third Hospital. TCGA, The Cancer Genome Atlas.

Supplementary Fig.S4: ROC curves and the corresponding AUCs for 3-year and 5-year DFS. a) ROC curves and the corresponding AUCs for 3-year DFS of each model in PUTH cohort. The confidence band shows AUC ± sd for the averaged ROC curves. b) ROC curves and the corresponding AUCs for 5-year DFS of each model. Abbreviations: DFS, disease-free survival. PUTH, Peking University Third Hospital.

Supplementary Fig.S5: Distribution of survival time for patients in the external sets. a) Distribution of survival time for patients in MCO cohort. b) Distribution of survival time for patients in TCGA cohort. Abbreviations: DSS, disease-specific survival. OS, overall survival

Supplementary Fig.S6: Multivariate Cox regression analyses between deep learning-predicted risk and clinicopathological variables. a) Multivariate Cox regression analysis for OS between deep learning-predicted risk, age, sex, and AJCC stage in MCO cohort. HRs and corresponding 95% conﬁdence intervals are presented. b) Multivariate Cox regression analysis for OS between deep learning-predicted risk, age, sex, and AJCC stage in TCGA cohort. c) Multivariate Cox regression analysis for DSS between deep learning-predicted risk, age, sex, and TNM stage in MCO cohort. d) Multivariate Cox regression analysis for DSS between deep learning-predicted risk, age, sex, and TNM stage in TCGA cohort. Abbreviations: DSS, disease-specific survival. HR, hazard ratio. MCO, Molecular and Cellular Oncology. OS, overall survival. TCGA, The Cancer Genome Atlas.

Supplementary Fig.S7: Multivariate Cox regression analyses between deep learning-predicted risk and clinicopathological variables in patients with non-metastatic colorectal cancer. a) Multivariate Cox regression analysis for DSS between deep learning-predicted risk, age, sex, and AJCC stage in MCO cohort. HRs and corresponding 95% conﬁdence intervals are presented. b) Multivariate Cox regression analysis for DSS between deep learning-predicted risk, age, sex, and AJCC stage in TCGA cohort. Abbreviations: DSS, disease-specific survival. HR, hazard ratio. MCO, Molecular and Cellular Oncology. TCGA, The Cancer Genome Atlas.

Supplementary Fig.S8: **Kaplan–Meier curves for DSS across different chemotherapy status or chemotherapy regimens in patients with stage II colorectal cancers.** a) Kaplan–Meier curves for DSS across different chemotherapy status in low-risk patients with stage II colorectal cancers. b) Kaplan–Meier curves for DSS across different chemotherapy status in high-risk patients with stage II colorectal cancers. c) Kaplan–Meier curves for DSS across different chemotherapy regimens in low-risk patients with stage II colorectal cancers. d) Kaplan–Meier curves for DSS across different chemotherapy regimens in high-risk patients with stage II colorectal cancers.

Supplementary Fig.S9: Guideline-based risk restratification of patients with stage III colorectal cancer in the MCO cohort. a) Kaplan-Meier curves for DSS across risk groups of patients with stage III colorectal cancer. Patients with pN2 or pT4 are classified as high risk. b) Guideline-based low-risk patients are restratified into low-risk and high-risk groups by deep learning-derived risk. c) Guideline-based high-risk patients are restratified into low-risk and high-risk groups by deep learning-derived risk. Abbreviations: DSS, disease-specific survival. HR, hazard ratio. MCO, Molecular and Cellular Oncology.

Supplementary Fig.S10: Integrating deep learning-derived risk with T stage and N stage for risk restratification of patients with stage II and III colorectal cancers in the MCO cohort. a) Patients with stage II colorectal cancer are restratified into low-risk, medium-risk, and high-risk groups by combining deep learning-derived risk and pathological T stage. b) The 5-year survival rates for DSS across low-risk, medium-risk, and high-risk groups. c) Kaplan-Meier curves for DSS across risk groups of patients with stage II colorectal cancer. d) Patients with stage III colorectal cancer are restratified into low-risk, medium-risk, and high-risk groups by combining deep learning-derived risk and pathological N stage. e) The 5-year survival rates for DSS across low-risk, medium-risk, and high-risk groups. f) Kaplan-Meier curves for DSS across risk groups of patients with stage III colorectal cancer. Abbreviations: DSS, disease-specific survival. HR, hazard ratio. MCO, Molecular and Cellular Oncology.

Supplementary Fig.S11: Heatmap visualization of patients with high predicted scores from PUTH cohort, MCO cohort, and TCGA cohort, respectively. H&E-stained WSIs (left) and corresponding whole-slide attention heatmap (right) are presented, with red region indicating high attention intensity. Scale bar: 5 mm. Abbreviations: DSS, disease-specific survival. MCO, Molecular and Cellular Oncology. PUTH, Peking University Third Hospital. TCGA, The Cancer Genome Atlas.

Supplementary Fig.S12: Heatmap visualization of patients with low predicted scores from PUTH cohort, MCO cohort, and TCGA cohort, respectively. H&E-stained WSIs (left) and corresponding whole-slide attention heatmap (right) are presented, with red region indicating high attention intensity. Scale bar: 5 mm. Abbreviations: OS, overall survival. MCO, Molecular and Cellular Oncology. PUTH, Peking University Third Hospital. TCGA, The Cancer Genome Atlas.

Supplementary Fig.S13: Interpretability analyses of quantitation in TCGA cohort. a) Patches with the highest attention score from low-risk and high-risk cases and corresponding cell annotations (in TCGA cohort). Scale bar: 100 μm. b) Quantitative comparison of cell fraction in high-attention patches across risk groups (in TCGA cohort). P-values are obtained by t test. Abbreviations: TCGA, The Cancer Genome Atlas.

Supplementary Fig.S14: Expression of canonical marker genes for each major cell type.

Supplementary Fig.S15: Dimensionality reduction for myeloid cells and epithelials. a) UMAP dimensionality reduction of myeloid cells. b) UMAP dimensionality reduction of epithelials. Abbreviations: UMAP, Uniform Manifold Approximation and Projection.

Supplementary Fig.S16: Dimensionality reduction and percentage of each subset for T cells. a) UMAP dimensionality reduction of T cells. b) Bar plots showing the percentage of each T cell subset across risk group. Abbreviations: UMAP, Uniform Manifold Approximation and Projection.

Supplementary Fig.S17: Enrichment analysis among top 20 patches between the low-risk and high-risk sample. a) Bar plot showing significantly enriched KEGG and GO pathways in the low-risk sample (P1CRC). b) Bar plot showing significantly enriched KEGG and GO pathways in the high-risk sample (P5CRC).
